# Antibacterial and Anti-Quorum Sensing Properties of Silver Nanoparticles Phytosynthesized Using *Embelia ruminata*

**DOI:** 10.3390/plants13020168

**Published:** 2024-01-08

**Authors:** Neervana Rambaran, Yougasphree Naidoo, Farzana Mohamed, Hafizah Y. Chenia, Himansu Baijnath

**Affiliations:** 1 Biological Sciences Department, School of Life Sciences, University of KwaZulu-Natal, Westville Campus, Durban 4000, South Africa; naidooy1@ukzn.ac.za (Y.N.); baijnathh@ukzn.ac.za (H.B.); 2 Microbiology Department, School of Life Sciences, University of KwaZulu-Natal, Westville Campus, Durban 4000, South Africa; fzana101@gmail.com (F.M.); cheniah@ukzn.ac.za (H.Y.C.)

**Keywords:** *Embelia ruminata*, silver nanoparticles, green synthesis, antibacterial, quorum sensing

## Abstract

The rise in antibiotic resistance (AR) poses an imminent threat to human health. Nanotechnology, together with mechanisms such as quorum sensing (QS), which relies on communication between bacterial cells, may decrease the selective pressure for AR. Thus, this study aimed to investigate the effectiveness of silver nanoparticles (AgNPs) synthesized at room temperature (Rt) and 80 °C using *Embelia ruminata* leaf, stem-bark, and fruit extracts as antibacterial and anti-QS agents. The phytosynthesized AgNPs solutions were subjected to various characterization assays and assessed for their antibacterial activities. Quantitative QS assays were performed using *Chromobacterium subtsugae* CV017 and *Chromobacterium violaceum* ATCC 12472. Synthesized AgNPs were spherical-to-near-spherical in shape, poly-dispersed, and crystalline, with a size range of 21.06–32.15 nm. Fruit AgNPs showed stronger antibacterial activity than AgNPs from other plant organs against selected bacterial strains. In the QS assays, fruit 80 °C AgNPs demonstrated the most significant violacein inhibition in an assay performed using the short-chain acyl homoserine lactone CV017 biosensor, while the leaf and fruit Rt AgNPs demonstrated the most violacein inhibition in an assay performed using the long-chain acyl homoserine lactone ATCC 12472 biosensor. The investigations carried out in this study lay the groundwork for future innovative research into antibacterial and anti-QS strategies using *E. ruminata*.

## 1. Introduction

Antibiotic resistance (AR) constitutes a major global healthcare concern and poses a significant challenge [1]. The employment of nanotechnology as a novel therapeutic strategy to overcome AR is proving to be particularly effective. Furthermore, the green synthesis of silver metal nanoparticles (AgNPs) using plant extracts is gaining popularity due to their biocompatibility and eco-friendly properties [2]. These nano-phytocomposites are considered a rich source of phytochemicals that can act as reducing and stabilizing agents during synthesis [3,4]. The size, shape, structure, and dispersity of the resultant nanostructures play a crucial role in the physicochemical and biological attributes of the nanoparticles (NPs) [5,6]. Congruently, reaction parameters such as temperature and time can play key roles in controlling the growth, shape, and size of NPs [7]. Furthermore, the small size and relatively large surface area of AgNPs enable a more pronounced antibacterial effect, as the particles can not only interact with the surface of the bacterial membrane, but also penetrate the bacteria, causing cell death [8].

As an adjunct to finding novel strategies to combat antibiotic resistance, the focus of some work has shifted to targeting anti-virulence processes such as quorum-sensing inhibition (QSI). Bacterial quorum sensing (QS) is a form of cell-to-cell communication by which bacteria exchange intracellular and intercellular information, coordinate population behavior and regulate gene expression [9]. The QS process is mediated by small, diffusible signal molecules (autoinducers) that are generated by the bacteria and released into the extracellular environment [10]. In Gram-negative bacteria, QS signalling occurs via N-acyl homoserine lactones (AHL) produced by AHL synthase (LuxI proteins) [11]. The structure of AHLs includes two parts, a homoserine lactone ring and a variable acyl side chain. The length of the side chain determines whether the molecules are short-chain AHLs (C4–C8) or long-chain AHLs (C10–C18) [12]. The AHLs diffuse out of the cell into the extracellular environment. When a threshold is reached, they return to the cell and bind with the receptor LuxR to form a LuxR/AHL complex and activate expression of downstream target genes, including the genes that code for virulence factors [12].

QS inhibitors, especially natural products such as phytochemicals, are of particular interest. These substances target and disrupt QS in bacteria without inhibiting bacterial growth [13,14]. Resistance development is thus reduced because minimal selective pressure is exerted on the pathogen [15,16]. There is limited information on the inhibition of QS and biofilm formation using nanoparticles synthesized from plant organs, creating the need for further investigations. [15,17,18]. 

*Embelia ruminata* (E.Mey. ex A.DC.) Mez, (Primulaceae) is a scandent shrub or liana found along the east coast of South Africa [19]. A benzoquinone derivative (3-undecyl 2,5-dihydroxy, 1,4- benzoquinone) also referred to as embelin is the principal compound found in the genus *Embelia* Burm.f. [20]. Several investigations have reported on the pharmacological properties of embelin, which include antimicrobial [21], anticancer [22], anti-Alzheimer’s [23], anti-inflammatory [24], antioxidant [25], antidiabetic [26], neuroprotective [27], and antiviral [28] properties. 

In this study, AgNPs synthesized using aqueous *E. ruminata* leaf, stem-bark, and fruit extracts were characterized using UV-visible spectroscopy, high-resolution transmission-electron microscopy (HR-TEM), selected-area electron diffraction (SAED), energy-dispersive X-ray analysis (EDX), Fourier-transformed infrared spectroscopy (FTIR), and zeta-potential analyses. Additionally, the study aimed to optimize the temperature and duration of the AgNPs fabrication process. Subsequently, the potential of *E. ruminata* AgNPs extracts as antibacterial agents was assessed using a panel of Gram-negative and Gram-positive bacterial species.

To assess their anti-QS properties, AgNPs phytosynthesized from *E. ruminata* were tested for their ability to inhibit the QS process of two Gram-negative biosensor strains, *Chromobacterium subtsugae* CV017 and *Chromobacterium violaceum* ATCC 12472. Both strains produce an easily detectable, quantifiable, water-insoluble purple pigment called violacein through the AHL QS process [29]. Thus, the ability of the synthesized nanoparticles to alter the production of violacein by the *Chromobacterium* species biosensors was evaluated by the loss of pigmentation, which was used as an indicator of QSI [30]. *C. subtsugae* CV017 produces short-chain (C4–C6) AHL signals [31], while *C. violaceum* ATCC 12472 produces long-chain (C10+) AHL signals [32]. Broad-spectrum QS inhibitors that disrupt the QS pathways of both long- and short-chain AHL signals are desirable, as they can limit pathogenicity and virulence over an extended range of bacterial strains [33]. The potential of AgNPs synthesized using the extracts of *E. ruminata* as broad-spectrum QS inhibitors was thus investigated.

## 2. Material and Methods

### 2.1. Collection and Preparation of Plant Material

The leaves, stem-bark, and fruit of *E. ruminata* were collected from Reservoir Hills, Durban, KwaZulu-Natal, South Africa (29°48′26.7″ S 30°55′43.9″ E), from February to May 2019 [34]. The species identity was confirmed using taxonomic keys, and a voucher specimen (Rambaran 1, accession number 18256) was deposited in the Ward Herbarium, University of KwaZulu-Natal, South Africa [34]. Plant samples were separated, air-dried, pulverized to a fine powder, and stored in airtight bottles [34]. For extraction, 20 g of the powdered plant material was added to 200 mL of deionized water in an Erlenmeyer flask and incubated in a water bath at 60 °C for 30 min. The resultant solution was cooled to room temperature (23 ± 2 °C), filtered using Whatman No.1 filter paper, and stored at 4 °C until further use. 

### 2.2. Synthesis of Silver Nanoparticles (AgNPs) 

A 5 mL aliquot of leaf, stem-bark, or fruit extract of *E. ruminata* was added to 45 mL of 1 mM of silver nitrate (AgNO_3_) solution for the bioreduction process [35]. The reaction mixture was monitored visually for a change in color at regular intervals. To investigate the effect of temperature, samples were incubated for 1 h at the experimental temperatures, i.e., at room temperature (Rt) (23 ± 2 °C) and at 80 °C, in a water bath. Thereafter, all samples were kept in the dark at room temperature for 24 h. The reaction mixtures were centrifuged at 10,000 rpm for 20 min at 4 °C using a Beckman Coulter Avanti J-E centrifuge. Supernatants were discarded, and pellets were washed three times with distilled water and centrifuged again. The purified pellets were transferred to glass vials, dried at 50 °C, and stored at room temperature until further use [36]. 

### 2.3. Characterization of Biosynthesized E. ruminata AgNPs

#### 2.3.1. UV-Visible Absorbance Spectroscopy

The optical absorption at different time intervals (0 min, 60 min, and 24 h) was analyzed using a Shimadzu UV-2600 spectrophotometer with a resolution of 1 nm and a wavelength range of 200–800 nm [37]. 

#### 2.3.2. High-Resolution Transmission-Electron Microscopy (HR-TEM) Analysis

A drop of sonicated AgNPs was placed onto carbon-coated copper grids lined with formvar and was allowed to dry by evaporation for 20 min at Rt (23 ± 2 °C). The structural characterization and the selected-area electron diffraction (SAED) patterns of AgNPs were acquired using a JEOL 2100 HRTEM (Tokyo, Japan) with an accelerating voltage of 200 kV [35]. The size of the nanoparticles was analyzed using iTEM (Soft imaging system, Germany Version 5.0). The elemental composition of the samples was determined using energy dispersive X-ray (EDX) analysis (Inca software coupled with an Oxford X-Max 80 mm detector, Oxford, UK). 

#### 2.3.3. Determination of Zeta Potential 

Nanoparticle tracking analysis (NTA) was used to evaluate the zeta (ζ) potential. A 1:1000 dilution of each sample in double-distilled water was analyzed using a Nanosight NS500 (Malvern Instruments, Worcestershire, UK, NTA 3.2 analytical software) equipped with a sCMOS camera with a laser wavelength of 430 nm. 

#### 2.3.4. Fourier-Transformed Infrared Analysis (FTIR) 

FTIR analysis of the crude *E. ruminata* extracts and their respective AgNPs was conducted using a Perkin-Elmer Spectrum 100 FTIR (Shelton, CT, USA) spectroscope with a scan range of 400 to 4000 cm^−1^ and a resolution of 4 cm^−1^ [38]. 

### 2.4. Antibacterial-Susceptibility Tests

Antibacterial-susceptibility assays were carried out using Kirby–Bauer disc diffusion with methods based on protocols previously published by Rambaran et al. [34]. Briefly, four Gram-negative and seven Gram-positive microorganisms were grown at 37 °C overnight on Mueller-Hinton (MH) agar plates. Inocula equivalent to McFarland standard 0.5 were swabbed onto the surfaces of MH agar plates [39]. Thereafter, blank discs impregnated with 100 and 200 μg of the AgNPs being tested were placed on the plates, and the plates were then incubated for 24 h at 37 °C. Ciprofloxacin (CIP5) and gentamicin (GN10) were used as the antibiotic controls. Following incubation, samples showing zone diameters ≥ 16 mm were regarded as showing strong antibacterial activity, zone diameters between 11–15 mm were regarded as having intermediate antibacterial activity, and zone diameters ≤ 10 mm were considered to possess weak antibacterial activity.

### 2.5. Qualitative Evaluation of Quorum-Sensing Inhibition

The QSI potential of *E. ruminata* AgNPs was investigated using the agar-overlay diffusion assay, following a protocol based on previously developed methodologies [34]. Briefly, *C. subtsugae* CV017 and *C. violaceum* ATCC 12472, were used as the indicator organisms [40]. Five milliliters of molten Lysogeny broth (LB) agar was inoculated with 150 μL of the *Chromobacterium* spp. biosensor strain to be tested, which was then grown overnight at 30 °C. The agar culture was then poured over the surface of pre-warmed LB agar plates and allowed to solidify. Next, blank discs were impregnated with 100 and 200 μg of the AgNPs being tested and placed on the surface of the agar plates. The plates were then incubated overnight at 30 °C. Discs impregnated with 100 and 200 μg of vanillin were used as the positive controls. After incubation, zone diameters were recorded. Opaque zone diameters indicated QS inhibition, while clear zone diameters indicated bactericidal activity. The AgNPs samples associated with opaque zone diameters ≥ 16 mm were considered to have strong potential for QS inhibition; zone diameters between 11–15 mm were considered to have intermediate for QS inhibition; and zone diameters ≤ 10 mm were considered to have weak potential for QS inhibition. 

### 2.6. Quantitative Quorum Sensing Inhibition

In this assay, 100 μL of the tested *Chromobacterium* spp. strains were cultured in 3 mL of LB medium and incubated at 30 °C with increasing concentrations of each AgNPs extract at 0, 20, 40, 80, 160, and 320 μg/mL. Bacterial growth (optical density (OD)_600 nm_) and violacein production (OD_560 nm_) were determined following an overnight incubation. One mL of the treated and untreated overnight cultures of *Chromobacterium* were centrifuged (Labnet Prism Microcentrifuge) at 10,000 rpm for 10 min to precipitate insoluble violacein. The pellet was resuspended in 1 mL of DMSO [37,38], centrifuged (10,000 rpm for 10 min), and quantified at OD_560 nm_. The following formula was used to calculate percentage of violacein inhibition (VI) [39,41].

% violacein inhibition = (control OD_560 nm_ − test OD_560 nm_/control OD_560 nm_) × 100 (1)

Phytosynthesized AgNPs at any concentration that resulted in VI ≥ 50% and growth inhibition (GI) ≤ 40% were considered QS inhibitors, while a VI ≥ 50% and GI > 40% indicated bactericidal rather than QSI activity.

### 2.7. Statistical Analysis

Statistical analyses were conducted using SPSS versions 26 and 27. The significance of the effect of temperature and time on synthesis, as well as the differences in the mean values of VI between extracts and between concentrations, were determined by one-way analysis of variance (ANOVA), which was followed by Tukey’s honest significant difference (HSD) post hoc test using a 95% confidence interval, with *p* ≤ 0.05 considered significant [10]. 

## 3. Results and Discussion

**Figure 1:**
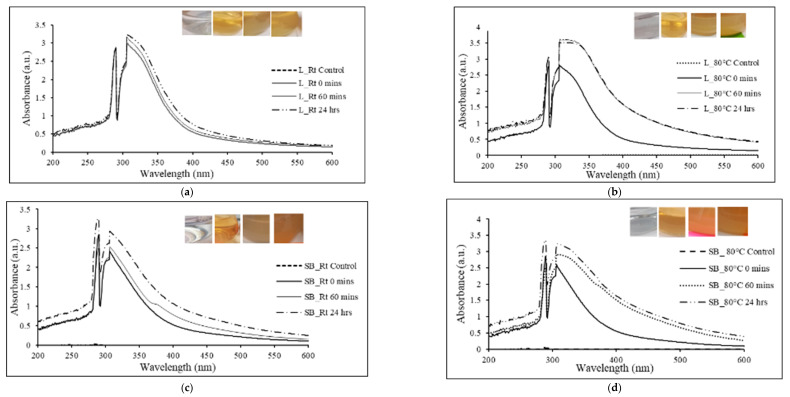

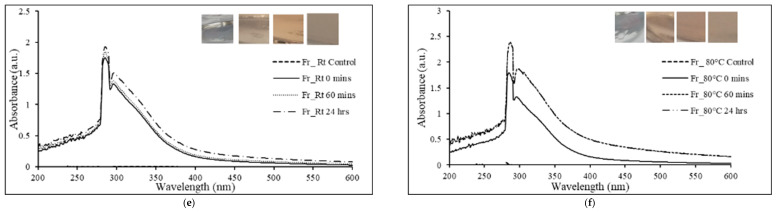
UV-vis absorption of aqueous *Embelia ruminata* silver nanoparticles (AgNPs) biosynthesized at room temperature and at 80 °C at 0 min, 60 min, and 24 h. Insets (from left to right) show the color changes observed during the biosynthesis of AgNPs at different time intervals (control, 0 min, 60 min, and 24 h). (**a**) L_Rt = leaf, room temperature; (**b**) L_80 °C = leaf, 80 °C; (**c**) SB_Rt = stem-bark, room temperature; (**d**) SB_80 °C = stem-bark, 80 °C, (**e**) F_Rt = fruit, room temperature; and (**f**) F_80 °C = fruit, 80 °C.

It has been documented that the reduction of silver ions results in a characteristic color change in the aqueous medium due to the excitation of the surface plasmon resonance (SPR) on the synthesized particles [42]. In this study, the addition of silver AgNO_3_ to the experimental plant extracts produced a wide range of color changes (Figure 1a–f insets). Leaf Rt extracts changed from a bright golden color to a cloudy yellow. Correspondingly, leaf 80 °C extracts displayed a similar color change, with greater intensity after the 1 h incubation period. The stem-bark extracts changed from a bright golden color to a cloudy brownish hue, with the change being more prominent in the 80 °C extracts. The fruit extracts changed from a faint pink to a dull ash color. Overall, different extracts produced different colors, signifying the varying reduction of Ag^+^ to Ag.

The conversion of silver to nanostructures was monitored by UV-visible spectroscopy, and absorbance peaks between 290 and 320 nm were observed for the synthesized AgNPs. This result further confirmed the biofabrication of AgNPs in reaction mixtures (Figure 1a–f). Notably, the intensity of the SPR peaks (Figure 1a–f) increased in the extracts from the initial time to 60 min, to 24 h, signifying the presence of increasing concentrations of AgNPs [43,44]. Moreover, the intensity of the color changes in the reaction mixtures was directly proportional to the incubation time [45]. This finding may imply that the synthesis of AgNPs increased with time, as observed from the increase in the absorbance of the SPR band (λmax = 290 to 320 nm) [46]. Varghese et al. [47] suggested that an SPR peak at 290 nm indicates proteins capped around the AgNPs that act as stabilizing agents for AgNPs.

Regarding the synthesis temperatures, no significant difference was observed in the nanoparticle size for samples incubated at Rt versus 80 °C. This finding may indicate that *E. ruminata* extracts developed nanocomposites of this size at Rt, without the effect of increased temperatures. Previous studies have shown that smaller nanoparticles are produced at higher temperatures of 80–90 °C [48]. Conversely, it has been noted that stable biosynthesis of plant extract-mediated AgNPs occurs at diverse temperatures ranging from 25 to >40 °C, with room temperature being preferred, as room temperature allows the formation of spherical AgNPs with single surface plasmons at low wavelengths [49]. It has been observed that higher biosynthesis temperatures yield larger nanoparticles under conditions of Ag^+^ precursor abundance [50]. In another study, it was postulated that increasing the temperature above 50 °C might lead to the degradation of compounds such as amino acids [51]. 

**Figure 2:**
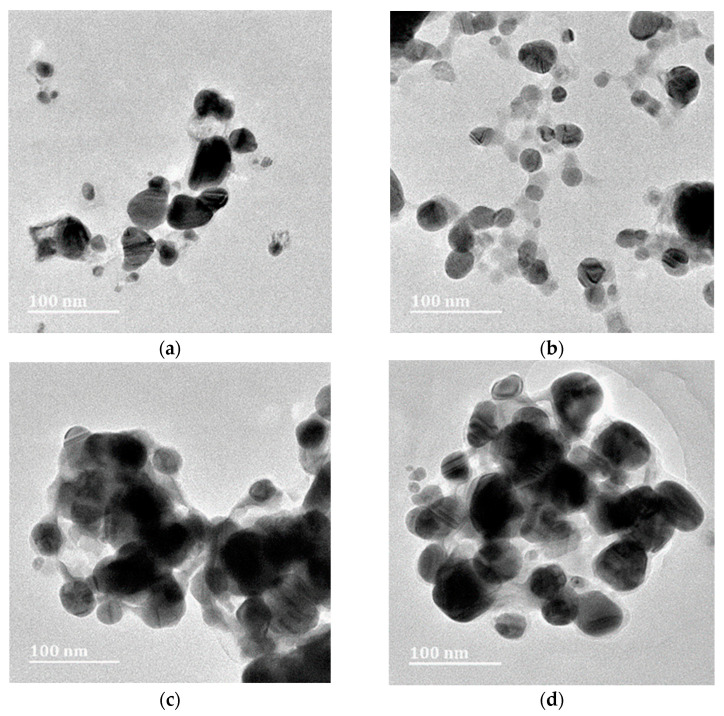

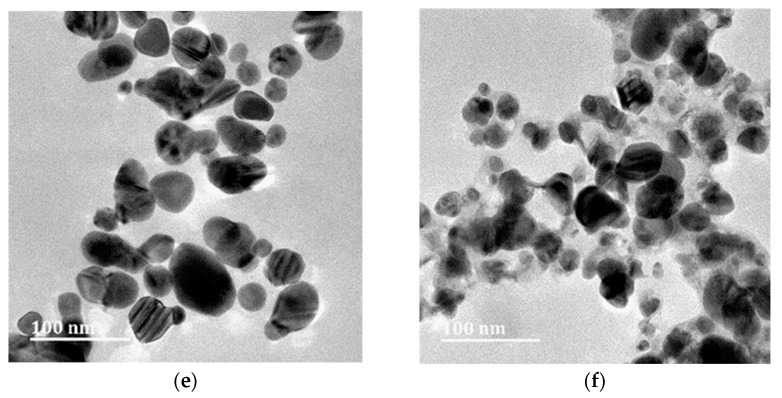
HR-TEM micrographs of silver nanoparticles (AgNPs)synthesized from *Embelia ruminata* extracts at room temperature (Rt) and 80 °C. Labels: (**a**) leaf, room temperature; (**b**) leaf, 80 °C; (**c**) stem-bark, room temperature; (**d**) stem-bark, 80 °C, (**e**) fruit, room temperature; and (**f**) fruit, 80 °C.

**Figure 3:**
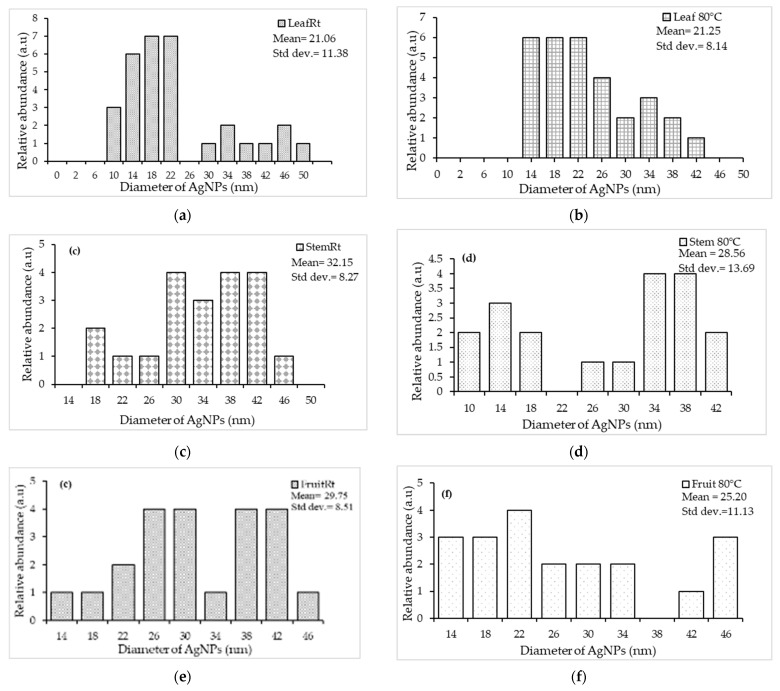
Nanoparticle-size-distribution histogram for silver nanoparticles (AgNPs) biosynthesized from *Embelia ruminata* extracts at room temperature (Rt) and 80 °C. Labels: (**a**) leaf, Rt; (**b**) leaf, 80 °C; (**c**) stem-bark, Rt; (**d**) stem-bark, 80 °C, (**e**) fruit, Rt; and (**f**) fruit, 80 °C.

**Figure 4:**
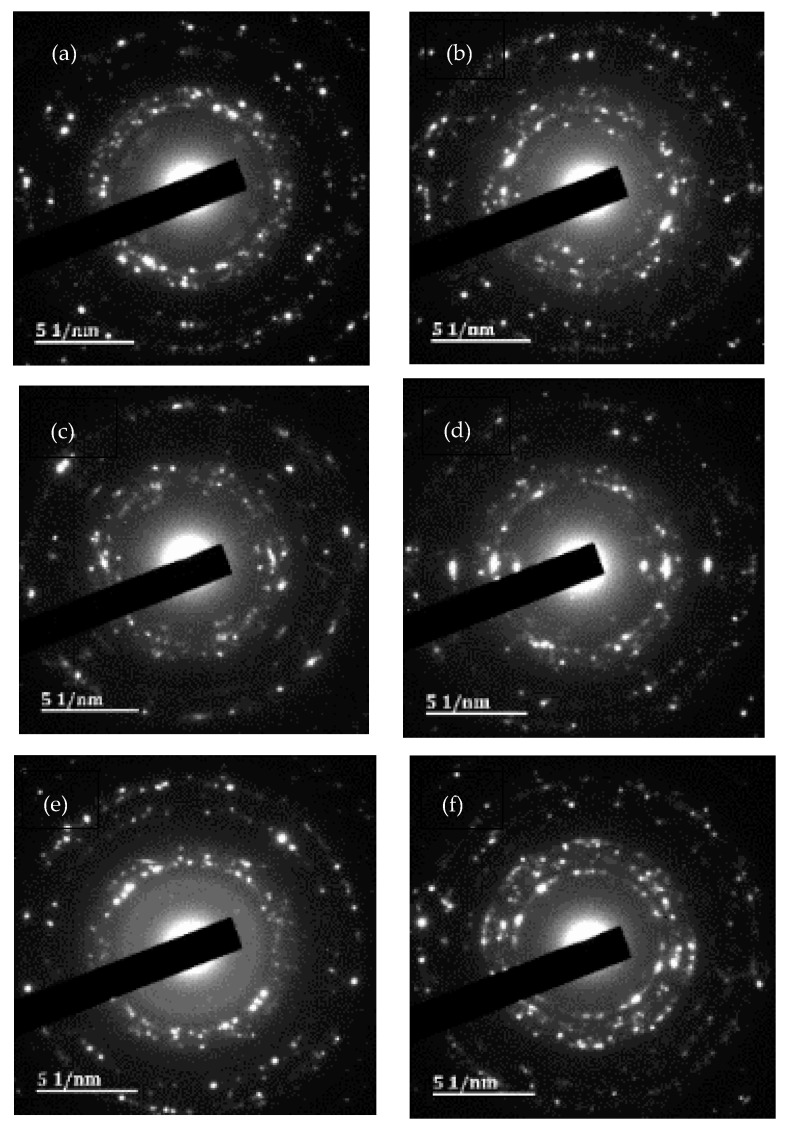
Selected-area electron-diffraction patterns of silver nanoparticles (AgNPs) biosynthesized from *Embelia ruminata* extracts at room temperature (Rt) and 80 °C. Labels: (**a**) leaf, Rt; (**b**) leaf, 80 °C; (**c**) stem-bark, Rt; (**d**) stem-bark, 80 °C, (**e**) fruit, Rt; and (**f**) fruit, 80 °C.

The HR-TEM results confirmed the presence of AgNPs with variable shapes and no notable differences among sample types were observed. Most of the AgNPs were spherical-to-almost-spherical (Figure 2a–f) and poly-dispersed, with a mean size range of 21.06–32.15 µm (Figure 3a–f). The EDX analysis showed a peak at 3 keV, which confirmed the presence of elemental silver ions [52]. The SAED results, together with d-spacing values, revealed that the synthesized AgNPs were polycrystalline, with a ring diffraction pattern that correlated to (111), (200) (220), and (222) (Figure 4a–f) [53,54]. The lattice plane (311) was not detected in the SAED patterns, possibly due to the rates of AgNPs synthesis [55]. Furthermore, there were no differences in the d-spacing of the crystal formation for samples synthesized at Rt versus 80 °C. These results agreed with HR-TEM data, which indicated that the formation of AgNPs was independent of temperature. Manikandan et al. [56] reported an average size of 30.2 ± 2 nm and the polycrystalline nature of AgNPs synthesized using the fruit of *Embelia ribes* Burm.f.

The NTA data showed a much larger size for AgNPs than did the HR-TEM analysis (Table 1). The disparity in the results may be due to the lower sensitivity of NTA; this method may have detected nanoparticles that were close to each other as larger particles [55]. Nanoparticles with a ζ-potential higher than +30 mV or lower than −30 mV [57] likely have sufficient repulsive force between the charged particles to attain better colloidal stability [58]. Conversely, a low zeta potential suggests particle aggregation and flocculation due to the van der Waals attractive forces that act upon the nanoparticles [59]. In the present study, all samples yielded a ζ-potential that ranged from −12.8–7.3 mV, indicating that AgNPs may incline towards lower stability and may have a greater tendency to aggregate. However, Joseph and Singhvi [58] stated that along with ζ-potential, other variables, such as material properties, presence of surfactants, and the solution chemistry, also influence the physical stability of the obtained nanosuspension. Their work suggests that zeta potential is not an absolute measurement of nanoparticle stability [60]. 

**Figure 5:**
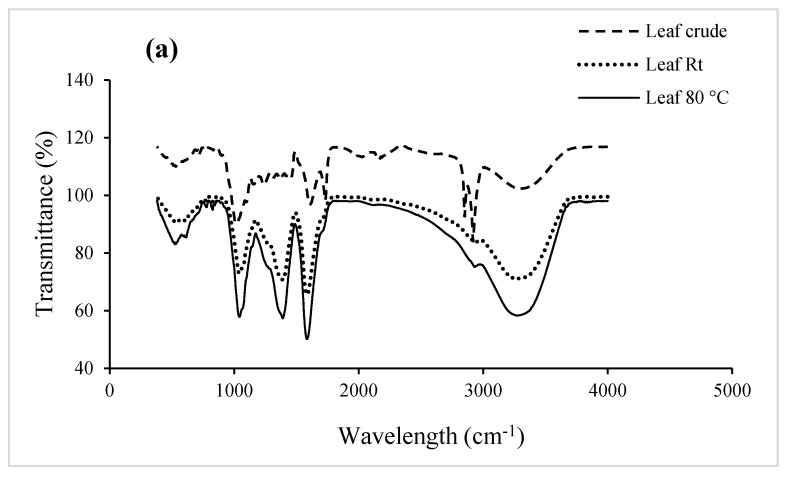

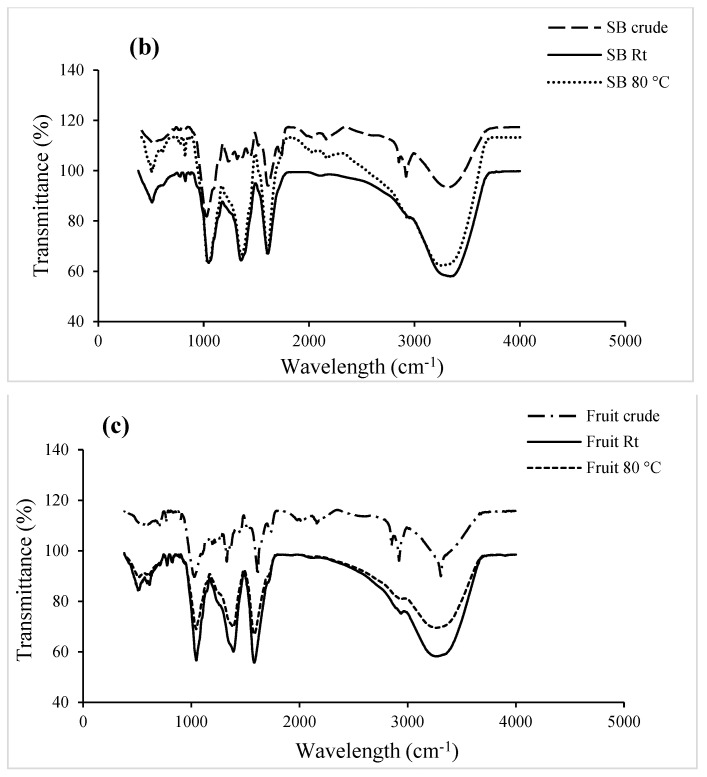
FTIR spectra of aqueous crude extracts and AgNPs of *Embelia ruminata* biosynthesized at room temperature (Rt) and 80 °C. Labels (**a**) leaf extract (**b**) stem-bark (SB) extract, and (**c**) fruit extract.

The FTIR absorption spectra of extracts before and after the reduction of Ag^+^ ions are presented in (Figure 5a–c). Absorption peaks in the 3600–3000 cm^−1^ range were observed for all biosynthesized AgNPs. These bands are characteristic of stretching vibrations of the O-H functional group in carboxylic acid and N-H stretching vibrations of amines in amino acids, peptides, and proteins [61,62]. Previous studies have indicated that hydroxyl groups serve as reducing agents and that carboxyl groups support the size and shape of nanoparticles [33,43]. New spectral peaks were observed at 1586.42, 1585.8, 1582.5, and 1582.73 cm^−1^ in leaf Rt and 80 °C AgNPs spectra, as well as in fruit Rt and 80 °C AgNPs spectra. This result could be attributed to the amide II N-H bending vibration coupled with C-N stretching at 1044.58, 1042.89, 1046.76, and 1045.23 cm^−1^ [63]. These absorption bands may indicate that proteins were interacting with the AgNPs [35]. Peak shifts were also noted at wavelengths of 1610.71 and 1606 cm^−1^ in the stem-bark Rt and 80 °C AgNPs. This could be attributed to a quinone compound, which has been shown to be responsible for the reduction of AgNO_3_ and for particle size [64].

In the antibacterial assays, the aqueous fruit AgNPs appeared to be most promising, with fruit Rt AgNPs showing intermediate antibacterial activity against sensitive *Escherichia coli* ATCC 25922 at 200 µg, multi-drug resistant *Pseudomonas aeruginosa* ATCC 27853 at 200 µg and *Staphylococcus. epidermidis* ATCC 12228 at 100 and 200 µg (Table 2). Similarly, fruit 80 °C AgNPs demonstrated intermediate activity against methicillin-resistant *Staphylococcus aureus* ATCC 33591 and ATCC 700698 at 200 µg, as well as against *S. epidermidis* ATCC 12228 at 100 and 200 µg. For comparison, in a study by Manikandan et al. [56], AgNPs synthesized using the fruit extracts of *E. ribes* showed intermediate and weak antibacterial inhibitory activity against *E. coli* and *S. aureus*, respectively. Additionally, in a previous study [34], *E. ruminata* chloroform fruit extracts showed greater inhibitory activity against methicillin-resistant *S. aureus* ATCC 700698 and vancomycin-resistant *Enterococcus faecalis* ATCC 51299 than the antibiotic controls ciprofloxacin and gentamicin [34]. Likewise, the fruit Rt and 80 °C AgNPs, at 100 and 200 µg, showed greater antibacterial activity against *S. aureus* ATCC 700698 than the antibiotic ciprofloxacin. Additionally, all AgNPs extracts at 200 µg showed greater antibacterial activity than gentamicin against *E. faecalis* ATCC 51299. 

Interestingly, apart from stem-bark RT AgNPs, all other AgNPs showed antibacterial activity against *S. epidermidis* at 200 µg (Table 2). Swamy et al. [65] demonstrated that ethanolic leaf extracts of *E. ribes* showed significant wound-healing activity compared to the standard skin ointment, framycetin. Additionally, the results from the current study support the ethnopharmacological use of *Embelia* leaves as a paste applied topically to cure skin infections [66]. 

Overall, the results of the antibacterial assays showed that AgNPs were more effective against Gram-positive bacteria than against Gram-negative bacteria. The outer membrane of Gram-negative bacteria poses a significant barrier to many compounds, including antibiotics. In comparison, the cell wall of Gram-positive bacteria is made up of several layers of peptidoglycan, enabling compounds to penetrate to varying degrees [67]. It was interesting to note that the AgNPs extracts showed greater antibacterial potential than the hexane, chloroform, and methanolic leaf, stem-bark, fruit, and seed extracts from previous studies [34]. The FTIR results showed a distinct OH group in all the AgNPs extracts. This finding was most prominent in the fruit 80 °C AgNPs. This result may signify the presence of functional phenol groups, which are known to exhibit antibacterial activity [68]. 

In the qualitative assessment of QSI using the short-chain AHL *C. subtsugae* CV017 biosensor, the stem-bark Rt, fruit Rt and 80 °C AgNPs demonstrated QSI at 100 to 200 µg, together with bactericidal activity (Table 3 and Supplementary Materials Figure S1a_1_–a_12_,b_1_–b_12_). At 200 µg, the leaf Rt AgNPs displayed QSI with killing, while the stem-bark 80 °C AgNPs demonstrated only QSI activity. In the assays using the long-chain AHL *C. violaceum* ATCC 12472 biosensor, all AgNPs displayed QSI activity at 200 µg (Table 3). The stem-bark Rt AgNPs showed only VI, while with the other AgNPs, VI was accompanied by growth inhibition. The objective of QSI is to disrupt quorum sensing; it is not aimed at killing the bacteria [13]. Thus, the most effective AgNPs were the stem-bark 80 °C and Rt AgNPs, which displayed QSI in the assays with the biosensors CV017 and ATCC 12472 at 200 µg, respectively, with no bactericidal effects.

**Figure 6:**
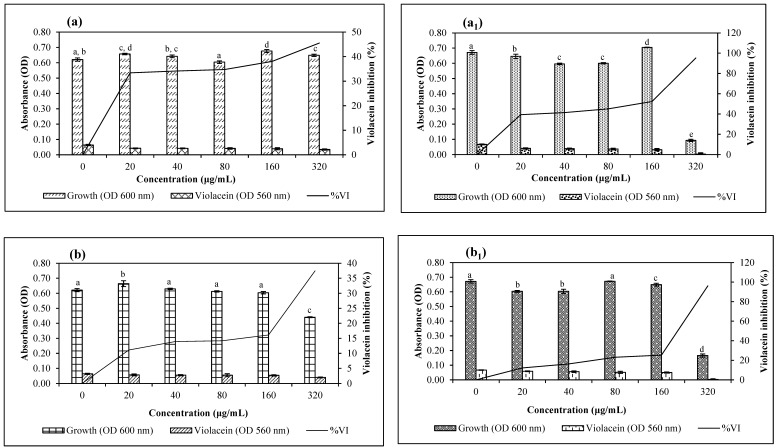

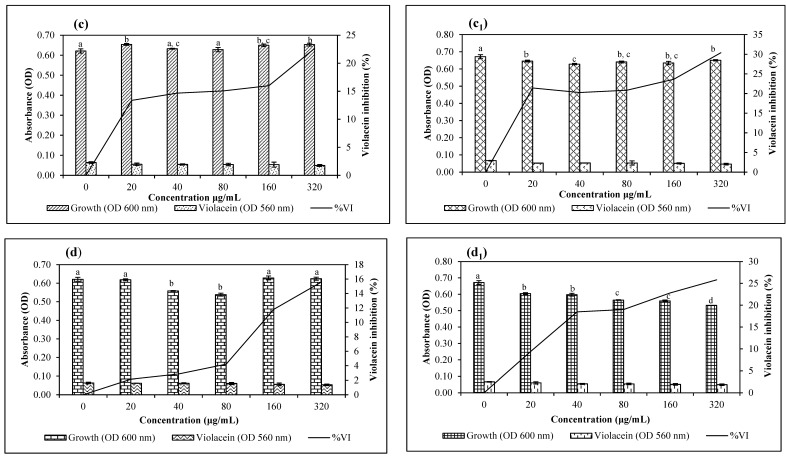

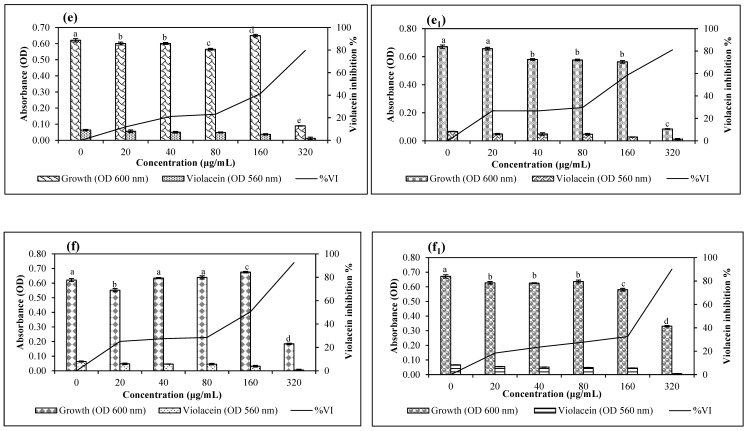
Quantitative analysis of the concentration-dependent inhibitory effects of silver nanoparticles (AgNPs) synthesized using aqueous *Embelia ruminata* extracts, on growth and violacein production by *Chromobacterium* spp. CV017 and ATCC 12472. Labels (**a**,**a_1_**) = Leaf Rt, (**b**,**b_1_**) = Leaf 80 °C, (**c**,**c_1_**) = Stem-bark Rt, (**d**,**d_1_**) = Stem-bark 80 °C, (**e**,**e_1_**) = Fruit Rt, (**f**,**f_1_**) = Fruit 80 °C using biosensors *Chromobacterium subtsugae* CV017 and *Chromobacterium violaceum* ATCC 12472, respectively. The average of three triplicate independent experiments and standard deviations are shown. Significance at *p* ≤ 0.05 indicated by different alphabetic letters.

In the quantitative violacein-inhibition assay, QSI was considered noteworthy when the % VI was ≥50% and the % GI was ≤40% (Figure 6a–f,a_1_–f_1_). Using the CV017 biosensor, fruit Rt AgNPs were bactericidal at 320 µg/mL with GI of 85.59% and an IC_50_ of 4.97 µg/mL (Figure 6e). The fruit 80 °C AgNPs (IC_50_ of 4.32 µg/mL) demonstrated VI of 50.47% at 160 µg/mL but were bactericidal at 320 µg/mL, with a GI of 70.27% (Figure 6f). This biosensor produces short-chain AHL signals, so inhibition indicates interference with short-chain AHL-based QS.

According to assays with the ATCC 12472 biosensor, the leaf and fruit Rt AgNPs with IC_50_ values of 3.8 and 4.39 µg/mL demonstrated QS inhibition ≥ 50% at 160 µg/mL with no growth inhibition and 16.20% GI (Figure 6a_1_ and e_1_), respectively. However, bactericidal activity was observed at 320 µg/mL with GI of 86.10 and 87.41%, respectively. This biosensor produces long-chain AHL signals, so inhibition indicates interference with long-chain AHL-based QS. 

In contrast the qualitative QSI tests, the AgNPs of *E. ruminata* had a more pronounced effect against long-chain than short-chain autoinducers. Studies have shown that NPs can form conjugates with phytocompounds that bind competitively to AHL receptor sites, thereby suppressing the QS circuit [69,70]. Thus, it is possible that the phytocompounds of *E. ruminata* AgNPs were able to bind to receptor sites, displacing AHL more effectively in the long-chain than the short-chain biosensor strain. 

According to previous studies, the hexane, chloroform and methanolic extracts of the leaf, stem-bark, fruit, and seed performed better in the QSI assays than did the AgNPs extracts [34]. This result may be due to the solubility of phytocompounds in the extracting solvent. Embelin, the principal compound of the genus *Embelia*, has a lipophilic affinity [71,72]. It is possible that embelin and other lipophilic compounds were not soluble in the aqueous extraction process for Ag nanosynthesis [73]. This insolubility may have influenced the better performance of the crude plant extracts compared to the AgNPs extracts as QS inhibitors. Furthermore, it is possible that the phytocompounds involved in AgNPs reduction and capping were not significant QSI agents. 

Statistical analyses showed that the mean values of violacein inhibition and thus QSI among AgNPs were greater than expected by chance and demonstrated a significant difference at *p* ≤ 0.05. Dwivedi and Singh [74] found that embelin isolated from *E. ribes* could inhibit biofilm formation by *Streptococcus mutans*, possibly by disrupting the QS pathway. Leema et al. [75] extrapolated from their studies that the antibacterial properties of the embelin-synthesized AgNPs were enhanced compared to the pure embelin extracts. Following this trend, further studies involving the isolation of principal compounds from *E. ruminata*, as well as exploring a range of extracting solvents and temperature gradients to synthesize AgNPs, may result in the derivatization of valuable QSI and biofilm-inhibitory compounds. 

## 4. Conclusions

The green synthesis of AgNPs using *E*. *ruminata* extracts was achieved and confirmed by the various characterization methods employed in this study. Additionally, the results indicated that the formation of AgNPs occurred at Rt, i.e., independent of elevated temperatures. Furthermore, the data suggest that the fruit AgNPs extracts exhibited the most promise as antibacterial agents. Likewise, in the quantitative QSI analyses, fruit 80 °C AgNPs demonstrated the most significant VI according to the result of the assays with the short-chain AHL CV017 biosensor, while the leaf and fruit Rt AgNPs demonstrated the most noteworthy VI according to the result of the assays with the long-chain acyl AHL ATCC 12472 biosensor. Regarding broad-spectrum QS inhibition, none of the AgNPs extracts exhibited the potential to disrupt the QS pathways of both long and short chain AHL signals. Despite this drawback, future research exploring the fractionation of pure compounds could yield phytochemicals with broad-spectrum QS inhibitory potential. Thus, this study has created the foundation for prospective studies and revealed the therapeutic potential of AgNPs derived from *E. ruminata*. Moreover, this research has demonstrated a novel strategy of using QS disruption to attenuate bacterial virulence and antibiotic resistance.

## Figures and Tables

**Table 1:** Comparison of particle size of *Embelia ruminata* silver nanoparticles (AgNPs) at room temperature (Rt) and 80 °C using HR-TEM, nanoparticle-tracking analysis, and zeta potential.

Samples	HR-TEM	NTA	
	Nanoparticle Size (nm) ± SD	Nanoparticle Size (nm) ± SD	ζ-Potential (mV) ± SD
Leaf Rt	21.06 ± 11.38	46.9 ± 1.4	−0.2 ± 0.2
80 °C	21.25 ± 8.14	102.0 ± 4.7	1.8 ± 0.7
Stem-Rt	32.15 ± 8.27	58.3 ± 16.1	−12.8 ± 1.4
80 °C	28.56 ± 13.69	65.9 ± 9.4	7.3 ± 0.2
Fruit Rt	29.75 ± 8.51	160 ± 19.5	−8.4 ± 1.0
80 °C	25.20 ± 11.13	198 ± 12.9	−0.2 ± 1.7

HR-TEM = High-resolution transmission-electron microscopy analysis, NTA = Nanoparticle Tracking Analysis, ζ = Zeta, and SD = Standard deviation.

**Table 2:** Antibacterial profile of *Embelia ruminata* silver nanoparticles (AgNPs) against selected Gram-negative and Gram-positive bacteria, showing zones of inhibition (mm).

Gram-Negative Pacteria									Gram-Positive Bacteria													
Sample	*E. coli* ATCC 25922		*E. coli* ATCC 35218		*K. pneumoniae* ATCC 700603		*P. aeruginosa* ATCC 27853		*E. faecalis* ATCC 29212		*E. faecalis* ATCC 51299		*S. aureus* ATCC 29213		*S. aureus* ATCC 33591		*S. aureus* ATCC 43300		*S. aureus* ATCC 700698		*S. epidermidis* ATCC 12228	
Concentration (µg)	100	200	100	200	100	200	100	200	100	200	100	200	100	200	100	200	100	200	100	200	100	200
NPs																						
Leaf Rt	0	**9**	0	**8**	0	**7**	0	**8**	**7**	**9**	**7**	**10**	**7**	**9**	**7**	**8**	**7**	**9**	**7**	**7**	**10**	**12**
Leaf 80 °C	0	0	0	**8**	**7**	**7**	0	**8**	**7**	**9**	**7**	**10**	**7**	**7**	**8**	**8**	**7**	**7**	**8**	**10**	0	**9**
Stem-bark Rt	0	0	0	0	0	0	0	0	0	0	0	**8**	0	0	0	0	**8**	**8**	0	0	0	0
Stem-bark 80 °C	0	**8**	0	0	0	0	0	0	0	**8**	0	**8**	0	0	0	0	0	0	0	0	**7**	**9**
Fruit Rt	**9**	**12**	**8**	**10**	0	**8**	**9**	**11**	**7**	**8**	**7**	**8**	**8**	**9**	**8**	**9**	**8**	**9**	**7**	**9**	**11**	**13**
Fruit 80 °C	0	0	0	**10**	0	**8**	**8**	**10**	**8**	**9**	**7**	**9**	**8**	**9**	**10**	**12**	**8**	**8**	**9**	**12**	**13**	**13**
Controls																						
Ciprofloxacin (CIP5)	30		37		26		32		33		38		23		22		23		6		28	
Gentamicin (GN10)	19		20		17		19		18		0		19		16		9		11		20	

Antibacterial activity classified as follows: ≤10 mm = weak, 11–15 mm = intermediate and ≥16 mm = strong.

**Table 3:** Qualitative evaluation of inhibitory effects of the aqueous silver nanoparticles (AgNPs) of *Embelia ruminata* leaf, stem-bark, and fruit on quorum sensing (QS), as determined by violacein production by *Chromobacterium subtsugae* and *Chromobacterium violaceum* biosensors.

	*C. subtsugae* CV017						*C. violaceum* ATCC 12472					
	100 μg			200 μg			100 μg			200 μg		
Extracts	Total Zone Diameter (mm)	Clear Zone Diameter (mm)	QSI Halo (mm)	Total Zone Diameter (mm)	Clear Zone Diameter (mm)	QSI Halo (mm)	Total Zone Diameter (mm)	Clear Zone Diameter (mm)	QSI Halo (mm)	Total Zone Diameter (mm)	Clear Zone Diameter (mm)	QSI Halo (mm)
Leaf RT	11	11	0	12	10	**2**	16	10	**6**	18	10	**8**
Leaf 80 °C	13	13	0	9	9	0	17	10	**7**	18	12	**6**
Stem-bark RT	14	9	**5**	16	9	**7**	12	8	**4**	13	8	**5**
Stem-bark 80 °C	0	0	0	**8**	0	**8**	13	8	**5**	13	9	**4**
Fruit RT	13	10	**3**	12	10	**2**	13	10	**3**	13	9	**4**
Fruit 80 °C	13	11	**2**	13	11	**2**	12	9	**3**	13	11	**2**
Control												
Vanillin	**11**	0	**11**	**11**	0	**11**	**9**	0	**9**	**9**	0	**9**

Light grey = bactericidal activity, medium grey = QSI + bactericidal activity, dark grey = QSI activity, QSI considered weak ≤10 mm, intermediate 11–15 mm and strong ≥16 mm, RT = room temperature (23 ± 2 °C).

## Data Availability

The data is contained within the manuscript and Supplementary Materials.

## Supplementary Materials

The following supporting information can be downloaded at: https://www.mdpi.com/article/10.3390/plants13020168/s1, Figure S1: Inhibitory effects of silver nanoparticles (AgNPs) biosynthesized using aqueous extracts of *Embelia ruminata* on violacein production by *Chromobacterium subtsugae* CV017 and *Chromobacterium violaceum* ATCC 12472.
