# Supplementary material for: Antibacterial and Anti-Quorum Sensing Properties of Silver Nanoparticles Phytosynthesized Using Embelia ruminata
Source: Plants (Basel). 2024 Jan 8;13(2):168. doi: 10.3390/plants13020168 (PMC10821412; doi:10.3390/plants13020168)
Supplement: Supplementary file 1 [file plants-13-00168-s001.zip › plants-2780734-supplementary.pdf]

plants

MDPI

*Supplementary Materials*

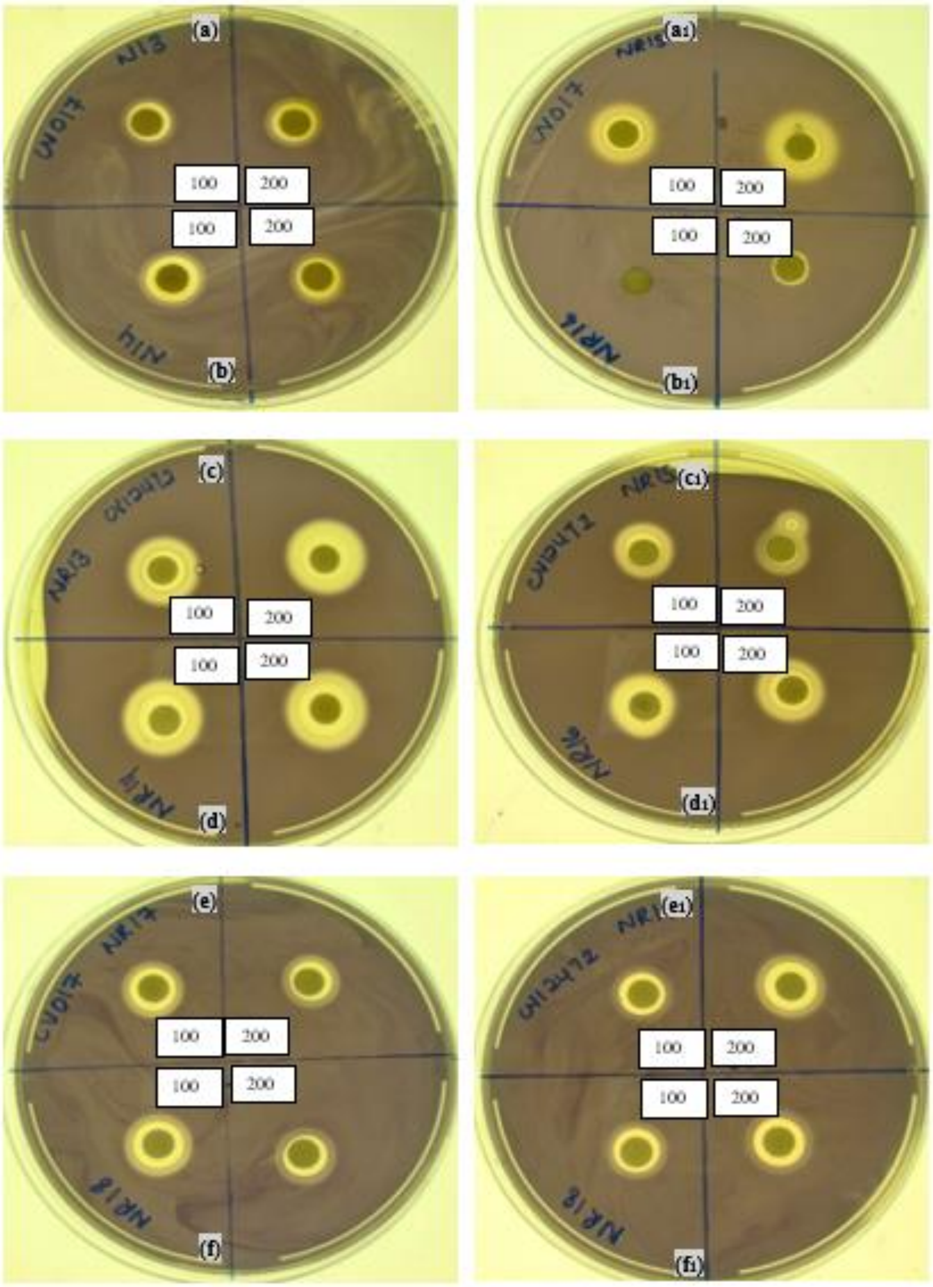


**Figure S1.** Inhibitory effects of silver nanoparticles (AgNPs) biosynthesized using aqueous extracts of *Embelia ruminata* on violacein production by *Chromobacterium subtsugae* CV017 and *Chromobacterium violaceum* ATCC 12472. Labels (a) and ($a_1$) = Leaf Rt, (b) and ($b_1$) = Leaf 80 °C, (c) and ($c_1$) = Stem-bark Rt, (d) and ($d_1$) = Stem-bark 80 °C, (e) and ($e_1$) = Fruit Rt, (f) and ($f_1$) = Fruit 80 °C using biosensors CV017 and ATCC 12472, respectively.
